# Classifying Characteristics of Opioid Use Disorder From Hospital Discharge Summaries Using Natural Language Processing

**DOI:** 10.3389/fpubh.2022.850619

**Published:** 2022-05-09

**Authors:** Melissa N. Poulsen, Philip J. Freda, Vanessa Troiani, Anahita Davoudi, Danielle L. Mowery

**Affiliations:** ^1^Department of Population Health Sciences, Geisinger, Danville, PA, United States; ^2^Department of Biostatistics, Epidemiology and Informatics, University of Pennsylvania, Philadelphia, PA, United States; ^3^Autism and Developmental Medicine Institute, Geisinger, Danville, PA, United States; ^4^Department of Biostatistics, Epidemiology and Informatics, Institute for Biomedical Informatics, University of Pennsylvania, Philadelphia, PA, United States

**Keywords:** machine learning, natural language processing, opioid-related disorders, substance use, supervised learning, deep learning

## Abstract

**Background:**

Opioid use disorder (OUD) is underdiagnosed in health system settings, limiting research on OUD using electronic health records (EHRs). Medical encounter notes can enrich structured EHR data with documented signs and symptoms of OUD and social risks and behaviors. To capture this information at scale, natural language processing (NLP) tools must be developed and evaluated. We developed and applied an annotation schema to deeply characterize OUD and related clinical, behavioral, and environmental factors, and automated the annotation schema using machine learning and deep learning-based approaches.

**Methods:**

Using the MIMIC-III Critical Care Database, we queried hospital discharge summaries of patients with *International Classification of Diseases* (ICD-9) OUD diagnostic codes. We developed an annotation schema to characterize problematic opioid use, identify individuals with potential OUD, and provide psychosocial context. Two annotators reviewed discharge summaries from 100 patients. We randomly sampled patients with their associated annotated sentences and divided them into training (66 patients; 2,127 annotated sentences) and testing (29 patients; 1,149 annotated sentences) sets. We used the training set to generate features, employing three NLP algorithms/knowledge sources. We trained and tested prediction models for classification with a traditional machine learner (logistic regression) and deep learning approach (Autogluon based on ELECTRA's replaced token detection model). We applied a five-fold cross-validation approach to reduce bias in performance estimates.

**Results:**

The resulting annotation schema contained 32 classes. We achieved moderate inter-annotator agreement, with F_1_-scores across all classes increasing from 48 to 66%. Five classes had a sufficient number of annotations for automation; of these, we observed consistently high performance (F_1_-scores) across training and testing sets for *drug screening* (training: 91–96; testing: 91–94) and *opioid type* (training: 86–96; testing: 86–99). Performance dropped from training and to testing sets for *other drug use* (training: 52–65; testing: 40–48), *pain management* (training: 72–78; testing: 61–78) and *psychiatric* (training: 73–80; testing: 72). Autogluon achieved the highest performance.

**Conclusion:**

This pilot study demonstrated that rich information regarding problematic opioid use can be manually identified by annotators. However, more training samples and features would improve our ability to reliably identify less common classes from clinical text, including text from outpatient settings.

## Introduction

In 2020, 9.5 million Americans aged 12 years and older had misused opioids in the past year and 2.7 million had an opioid use disorder (OUD) (1). OUD is characterized by a loss of control of opioid use, risky opioid use, impaired social functioning, tolerance, and withdrawal, as defined by the *Diagnostic and Statistical Manual of Mental Disorders-5* (DSM-5). Opioid misuse and OUD have a host of negative impacts on individuals' health and quality of life, including risk of overdose and death. In 2020, overdose deaths reached a new high of 93,000; of these deaths, approximately 70,000 were attributable to opioids, including prescription opioids, heroin, and fentanyl (2). The opioid epidemic presents an urgent public health crisis that warrants innovative research strategies to identify those at risk for opioid-related morbidity and mortality.

### Opioid Use Disorder Research Using Electronic Health Records

Electronic health records (EHRs) have been widely used for population health research (3). Most studies rely upon structured data contained within EHRs—such as diagnostic codes, medication orders, or laboratory tests—to identify individuals with specific conditions. Regarding OUD, a review of studies through 2015 identified 15 algorithms developed to identify non-medical opioid use, the majority of which used medical claims data (4). Such algorithms that incorporate opioid prescriptions are particularly useful for identifying iatrogenic cases of OUD (stemming from prescription opioid dependence) (5). Given the underdiagnosis of OUD (6), structured EHR data has less utility for identifying OUD that may have arisen through illicit opioid use. The historic underdiagnosis of OUD may be due to several factors, including uncertainty in diagnosing the condition by providers lacking specialty training, as well as stigma that leads providers to avoid assigning diagnostic codes for opioid misuse or patients to hide their condition (6, 7). Unstructured data contained within EHRs, including clinical narratives within medical encounter notes, document signs and symptoms of OUD as well as social risks and behaviors that may not be captured with diagnostic codes, providing a useful source of data that can enrich structured EHR data.

### Framework for Developing Natural Language Processing Tools

Efficiently synthesizing information from clinical text requires automated information extraction techniques such as natural language processing (NLP). An important first step to NLP is the development of a rigorous annotation process, which is critical to the reliability and performance of the NLP system (8). The standard approach to annotation includes multiple annotators reviewing and marking the same data and computing agreement across annotators, generally measured by inter-annotator agreement (IAA). IAA provides an indication of the difficulty and clarity of a task. To develop a high-quality corpus of annotated text, annotators follow a set of guidelines to ensure the process is consistent and objective (8).

Modern NLP methods include symbolic rules, machine learning, deep learning, and hybrid-based approaches. Validation processes are used to reduce biased performance estimates, particularly for studies with small sample sizes in which there is less statistical power for pattern recognition (9). Feature selection, cross-validation, and train/test split approaches have been shown to produce less biased performance estimates, even with a small sample (9). Performance of NLP tools are typically evaluated using measures of recall, precision, and F_1_-score (10).

### Natural Language Processing for Opioid Use Disorder Identification

Prior studies have utilized NLP to identify problematic opioid use (7, 11–15) and opioid overdose (16) from EHR and paramedic response documentation (17). However, several gaps remain in the development of NLP systems to identify problematic opioid use and OUD. Symbolic rule-based systems that rely on keyword lists, regular expressions, and term co-occurrence have been most commonly developed, such as nDepth^TM^ (11) and MediClass (16), among other tools (7, 12, 13). More contemporary NLP approaches remain limited, with only three previous studies having applied machine learning methods to identify opioid misuse (14, 15, 17). Of these studies, only Lingeman and colleagues (14) described details of their annotation process, with annotation performed by a single annotator. Lingeman and colleagues (14) also expanded beyond keywords such as “opioid abuse” to capture a greater range of opioid-related aberrant behaviors. However, other clinical, behavioral, and environmental factors linked to OUD documented in clinical notes, such as other substance use disorders, psychiatric co-morbidities, chronic pain, overdose, and social determinants of health (e.g., homelessness) could prove useful in characterizing OUD. Finally, prior studies have primarily been conducted among patients on long-term prescription opioids, e.g., as therapy for chronic pain, with one exception (15), missing opportunities to identify and study OUD related to illicit opioid use in the population.

Thus, studies are needed that utilize rigorous annotation approaches to inform NLP systems that include individuals who developed OUD through illicit opioid use and that draw upon additional information contained in clinical text to deeply characterize problematic opioid use. Such efforts could inform development of an NLP tool that would facilitate more accurate case finding in EHR data, bolstering a range of research on OUD, including epidemiologic, clinical, and genetic studies (18). Our long-term objective is to develop an NLP system that identifies and characterizes cases of OUD arising from both prescription and illicit opioid use to conduct EHR-based studies to understand biological, patient, provider, and community factors associated with OUD. Our short-term objectives in this study were to develop and apply an annotation schema to deeply characterize OUD, and to automate the schema using machine learning and deep learning-based approaches. Herein, we describe our annotation process and schema, and then present the results of two supervised classification approaches.

## Methods

We first developed an annotation schema to characterize problematic opioid use, identify individuals with potential OUD, and provide psychosocial context surrounding the condition. We applied the schema to clinical notes of de-identified patients with an OUD diagnosis. We then developed computational methods to automate the schema using machine and deep learning and evaluated the informativeness of features for predicting OUD in its contexts within sentences within hospital encounter documentation. The Geisinger and University of Pennsylvania Institutional Review Boards reviewed the protocol for this study and determined it met criteria for exempt human subjects research, as all data were fully de-identified.

### Study Population

Individuals included in this study came from the MIMIC-III Critical Care Database, a publicly-available, de-identified dataset that includes clinical data for roughly 60,000 patients with a hospital stay at Beth Israel Deaconess Medical Center in Boston, Massachusetts between 2001–2012 (19). From the MIMIC-III dataset, we downloaded discharge summaries from 762 patients who had an *International Classification of Diseases, version 9* (ICD-9) code related to OUD (304.00–304.03, 304.7, 304.70–304.73, 304.8, 304.81, 304.82, 304.83, 305.50–305.53, 965.00, 965.01, 965.02, 965.09, E850.0, E935.0).

### Annotation Schema Development

Initial development of the annotation schema included both deductive and inductive approaches to defining classes. We first drew upon prior research that used medical record review to identify OUD based on DSM-5 criteria (6), creating classes to reflect these criteria. We added to this initial set of classes by reading through discharge summaries and considering instances related to opioid and other drug use, as well as our knowledge of previously identified risk factors for OUD (e.g., psychiatric conditions). We then iteratively refined the initial schema through our first round of annotation of discharge summaries for five patients. We developed guidelines that defined each class and provided examples to ensure consistency between annotators. All authors were involved in the schema development.

**Figure 1:**
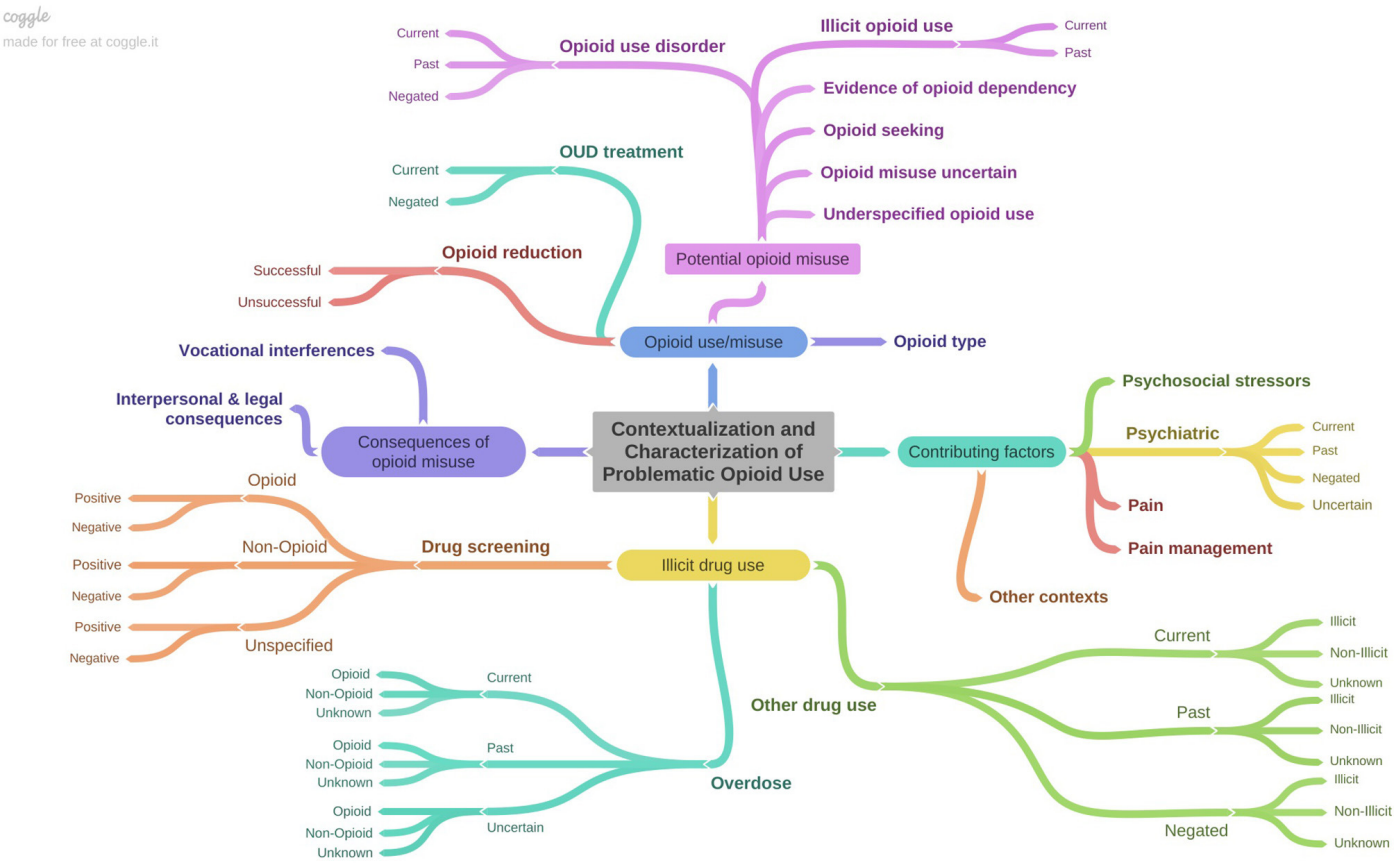
Depiction of annotation schema characterizing OUD-related information contained in discharge summaries. Figure created using Coggle (https://coggle.it/).

The final annotation schema represented a deep characterization of OUD-related information documented in clinical notes, with 32 classes related to problematic opioid use, factors contributing to opioid use/misuse, substance use, and consequences of opioid misuse (Figure 1; Supplementary Table 1). Several classes contained attributes (e.g., drug screening types and results). We included a class labeled *other contexts* to capture details in discharge summaries that were potentially relevant for OUD, but for which we had not defined a specific class (e.g., “altered mental status,” “counseled on drug use”). This class was largely intended to inform future changes to the annotation schema. We also included a patient-level assertion of OUD status, which was annotated at the level of each discharge summary rather than at the sentence level. This was made based on the clinical writer's assertion of OUD status rather than the annotators' assessment and was classified as positive, negative, uncertain, or not-specified. For example, a discharge summary in which the clinical writer noted that the patient abused heroin or was receiving methadone treatment at a drug treatment facility was classified as “positive,” whereas a summary in which the clinical writer made no comments indicating whether or not the patient had an opioid use disorder was classified as “not-specified.”

### Annotation Study

We leveraged an open-source text annotation tool called the extensible Human Oracle Suite of Tools (eHOST) (20) to annotate discharge summaries. Two authors (MP and PF) separately reviewed the full discharge summaries and annotated individual sentences for the same 40 patients over eight rounds of annotation (corpus 1). Annotation was completed at the sentence level, assigning full sentences to one or more relevant classes, with the exceptions of the class *opioid type*, for which we annotated phrases (i.e., the specific opioid name) and the patient-level OUD assertion. After completing a batch of five patients and their associated notes, we calculated F_1_-scores to capture IAA among classes and types for overlapping spans (IAA was not calculated for the first batch because this batch was primarily used to refine the annotation schema; annotations from these five patients were also not included in the automation study). Annotations were adjudicated with disagreements resolved through discussion with all study authors. Once the IAA was deemed sufficient to begin separate annotation work, the same two authors then annotated discharge summaries for a unique set of 30 patients each (total of 60 patients; corpus 2). We report the IAA agreement over each batch as well as the frequency distribution and highest IAA achieved for each class.

### Automation Study

#### Experimental Design

We randomly sampled patients with their associated annotated sentences and divided them into training (65%) and testing (35%) sets. The training set was used to generate and select the most informative features for predicting each class and reduce the likelihood of overfitting. To ensure the comparability of the training and testing sets, we evaluated class distributions between the two data sets.

#### Feature Generation and Selection

We leveraged the training set to generate and select features informative for training prediction models to classify sentences according to each class from the annotation schema. First, for each entry from the training dataset, we preprocessed the text to reduce case and add spaces around punctuation to best encode terms.

Next, we selected three open-source NLP systems/knowledge bases to encode semantic features from the annotated sentences: **Empath, Unified Medical Language System** (UMLS), and **ConText** (Table 1). We chose these approaches and systems to encode features based on their demonstrated informativeness in prior studies from the OUD literature [e.g., (7, 11, 14, 15)]. We applied Empath (21), a tool that draws connotations between words and phrases based on neural word embeddings from over 1.8 billion words of modern fiction, to generate semantic categories based on lay terms, including categories describing clinical, behavioral, and environmental factors such as pain, alcohol, crime, and family. We applied existing categories derived from Empath based on the reddit corpus, which captures common, rather than clinical, language to describe these concepts. However, Empath has broader coverage of terms related to these topics, at the expense of semantic precision. Therefore, we removed existing, built-in categories that did not capture accurate semantics in the clinical text (e.g., the category of “heroic” spuriously encoded “heroine,” a misspelling of “heroin”). To overcome limitations in coverage of relevant concepts by Empath, we added novel categories, including “opioid,” “dosage,” “overdose,” “withdrawal,” “psychiatric,” and “substance abuse.” Next, we leveraged scispacy to encode clinical concepts from the UMLS, a standardized vocabulary of biomedical concepts (22). The UMLS contains a robust terminology for clinical conditions (sign or symptom, disease or syndrome, mental or behavioral dysfunction) illicit and non-illicit drugs (clinical drug, pharmacologic substance), among other medical concepts mapped to concept unique identifiers (CUIs; e.g., “heroin” and “diamorphine” maps to “C0011892”). Finally, we applied the python version of the ConText algorithm to encode contextual information important for discerning historical from recent events, references to patients from references to family members, and negations from affirmed states (23, 24). We also encoded syntactic information including use of conjunctions, pseudo-negations, etc. Examples of features and their usage can be found in Table 1.

**Table 1:** Feature types used to train and test supervised classifiers, with examples of features and related annotation sentences.

**Feature group**	**Feature type**	**Example of feature**	**Example annotated sentence**
Semantic	Empath	substance_abuse	“**smokes** 3 ppd x many years”
	UMLS CUI	C0030049_oxycodone	“**oxycodone** for pain mgmt”
Contextual	ConText	definite_negated_existence	“**denies** using heroin”

We generated boxplot representations and applied an ANOVA to statistically compare the training and testing datasets based on the mean length of annotations (i.e., the number of words) in each class and the mean number of features per class for each feature type. We applied Chi-square feature selection to identify and retain only the most informative features for classifying each class. We graphed the frequency distribution of the reduced set of features by type and class. All graphs were generated using the R package.

#### Sentence Classification

We developed prediction models for classifying each sentence according to class using scikit-learn (25) and Autogluon version 0.3.1 (26–28), two machine learning and data science packages for developing prediction models for binary classification tasks. We trained and tested two supervised machine and deep learning classifiers—**logistic regression** and **Autogluon** (28)—to classify each sentence according to an OUD class.

Each algorithm was trained using the default settings, as described below. No hyperparameter tuning was carried out.

- **Logistic regression:** This classifier uses a sigmoid function defined by linear transformation of the features to find the best model to describe the relationship between the target variable (output) and a given set of features (inputs). The default parameters were penalty = “l2”, ^*^, dual = False, tol = 0.0001, C = 1.0, fit_intercept = True, intercept_scaling = 1, class_weight = None, random_state = None, solver = “lbfgs”, max_iter = 100, multi_class = “auto”, verbose = 0, warm_start = False, n_jobs = None, l1_ratio = None.
- **Autogluon:** We trained and tested TextPredictor, which fits a transformer neural network model using transfer learning from a pretrained model, ELECTRA (**E**fficiently **L**earning an **E**ncoder that **C**lassifies **T**oken **R**eplacements **A**ccurately). ELECTRA leverages a replaced token detection rather than masked learning models like BERT (**B**idirectional **E**ncoder **R**epresentations from **T**ransformers) (29) and has been shown to produce superior results to BERT given the same model size, data, and compute (28, 30). The pretrained model is an electra base discriminator with a learning rate decay of 0.90. Class predictions were output through two additional dense layers. Each classifier was trained using ten epochs and 150 iterations.

#### Validation and Performance Evaluation

For both supervised learning classifiers, we trained and tested prediction models for classes with at least 100 annotations in the training set. We did not evaluate the *other contexts* class because it was not meaningful for OUD characterization. Classes with fewer annotations were not included due to concerns about overfitting, which could result in less robust and poorly generalizable prediction models.

We implemented a cross-validation approach using both the training and testing folds in an effort to reduce the likelihood of producing biased performance estimates due to small sample sizes. We applied a five-fold cross-validation approach to train the prediction models on the training set, reporting the average performance across validation folds. The testing set was separated into 5-folds to provide an additional external validation of the prediction models generated by the training set. We computed the standard performance metrics of recall (sensitivity) and precision (positive predictive value) to evaluate how well-each classifier identified each class. We also computed F_1_-score—the harmonic mean between recall and precision—to select the classifier with the best performance (10). Training was optimized for F_1_-score. We report the means and 95% confidence intervals for the 5-fold cross-validation results.

## Results

We conducted an annotation and automation study for encoding OUD-related sentences from clinical texts.

### Annotation Study

**Figure 2:**
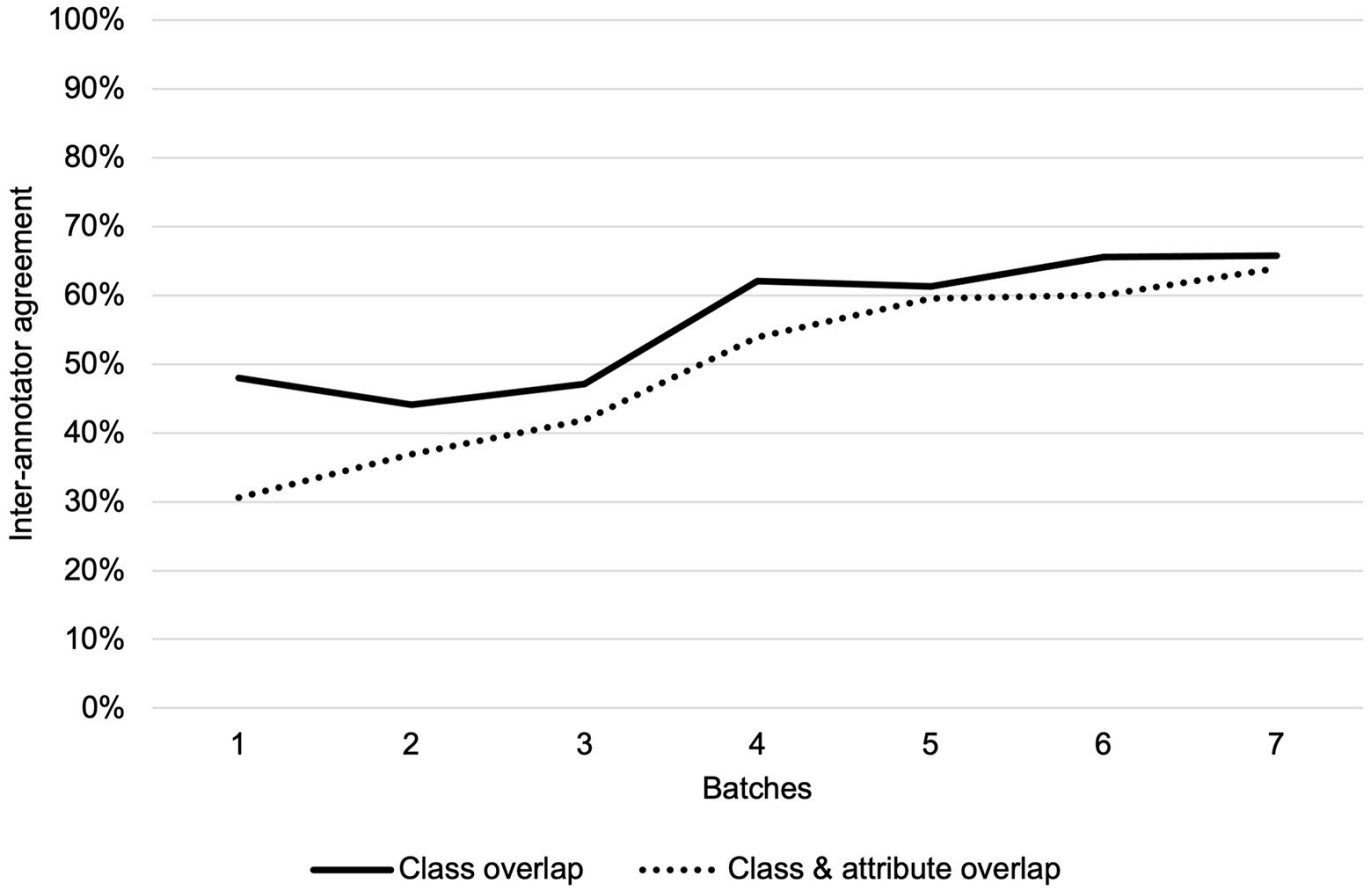
Inter-annotator agreement over seven annotation batches for class overlap and class and attribute overlap.

We adjudicated annotations for corpus 1 and two annotators separately annotated discharge summaries for corpus 2, for a total of 3,720 annotations within 138 total discharge summaries. Overall IAA (F_1_-score) for corpus 1 across all classes increased from 48% to 66% for the same class and from 31% to 64% for the same class and attribute over the seven annotation batches (Figure 2). The highest IAA achieved for patient-level assertion of OUD status was 67%. We observed low IAA for less prevalent classes and improving IAA for more prevalent classes (Table 2).

**Table 2:** Highest achieved IAA (based on class overlap) of each class, and frequency of use for each corpus of discharge summaries.

**Class**	**Highest IAA**	**Frequency**	
		**Corpus 1**	**Corpus 2**
Opioid type	88%	258	455
Illicit opioid use (current)	57%	25	62
Illicit opioid use (past)	100%	19	17
Opioid dependency	89%	21	14
Opioid seeking	0%	6	2
Opioid misuse uncertain	–	1	2
Underspecified opioid use	0%	8	17
OUD (current)	0%	9	18
OUD (past)	0%	2	2
OUD (negated)	0%	1	0
OUD treatment (current)	71%	35	36
OUD treatment (negated)	0%	0	1
Opioid reduction (successful)	100%	1	0
Opioid reduction (unsuccessful)	0%	0	0
Psychosocial stressors	67%	14	38
Psychiatric (current)	86%	98	162
Psychiatric (past)	0%	8	0
Psychiatric (negated)	100%	12	13
Psychiatric (uncertain)	0%	16	4
Pain	86%	43	113
Pain management	67%	92	182
Other contexts	40%	77	117
Other drug use (current)	69%	147	331
Other drug use (past)	62%	26	68
Other drug use (negated)	0%	38	54
Overdose (current)	100%	56	66
Overdose (past)	100%	3	6
Overdose (negated)	0%	2	0
Overdose (uncertain)	75%	18	27
Drug screening	83%	124	161
Vocational interferences	–	0	0
Interpersonal and legal consequences	0%	5	0

### Automation Study

#### Feature Generation and Selection

**Figure 3:**
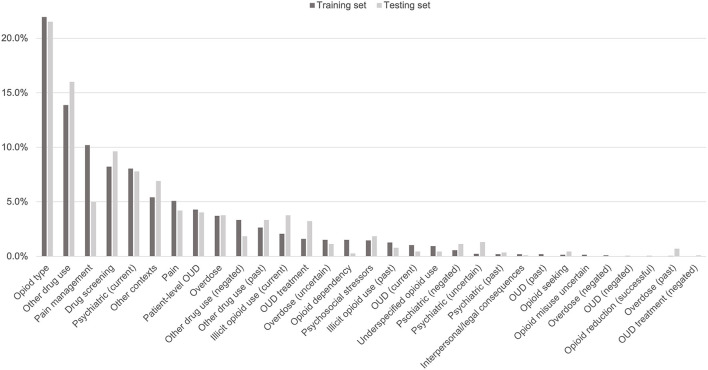
Distribution of classes in testing and training data sets.

**Figure 4:**
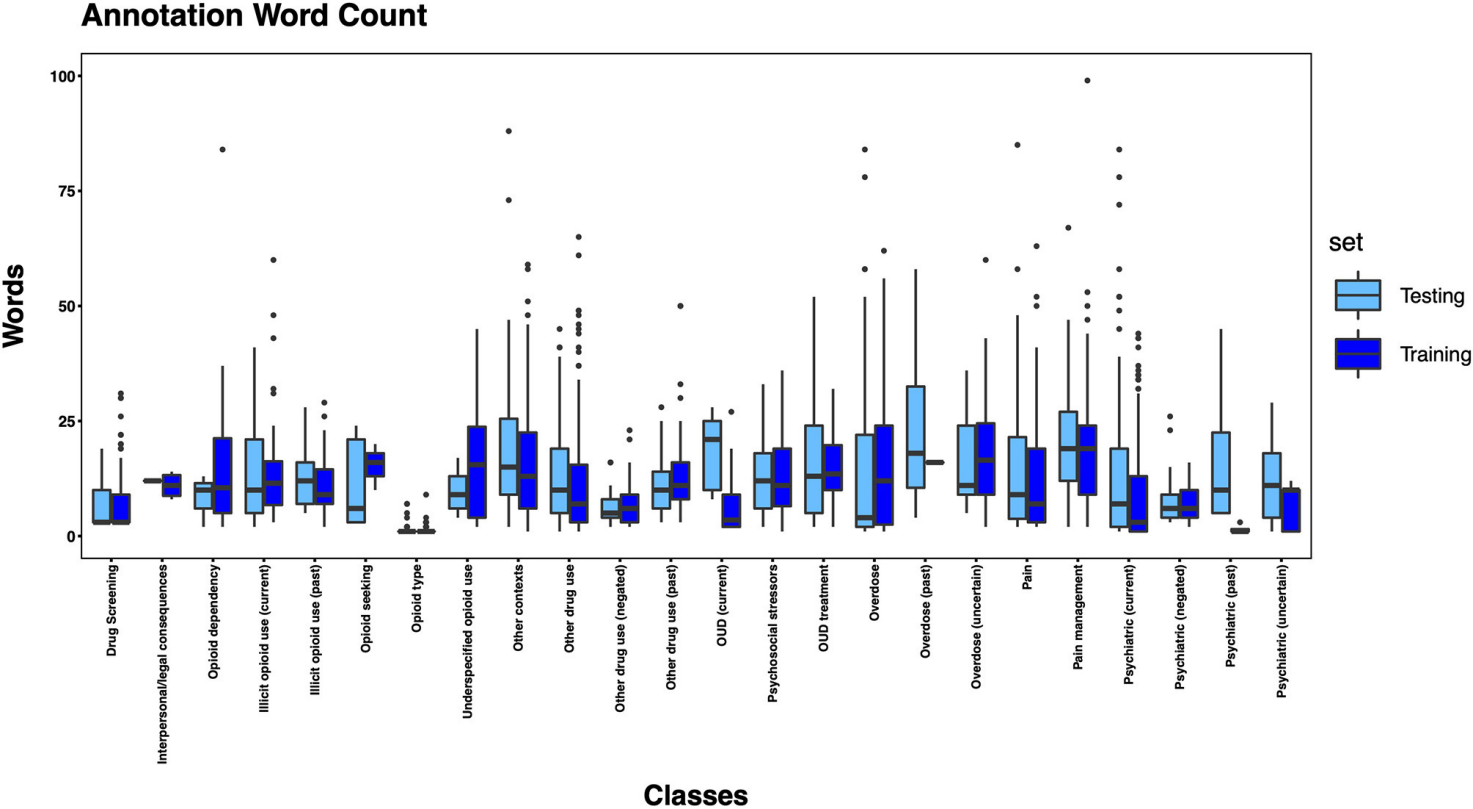
Boxplot of the mean count of words per annotation, by class, comparing training and testing sets.

**Figure 5:**
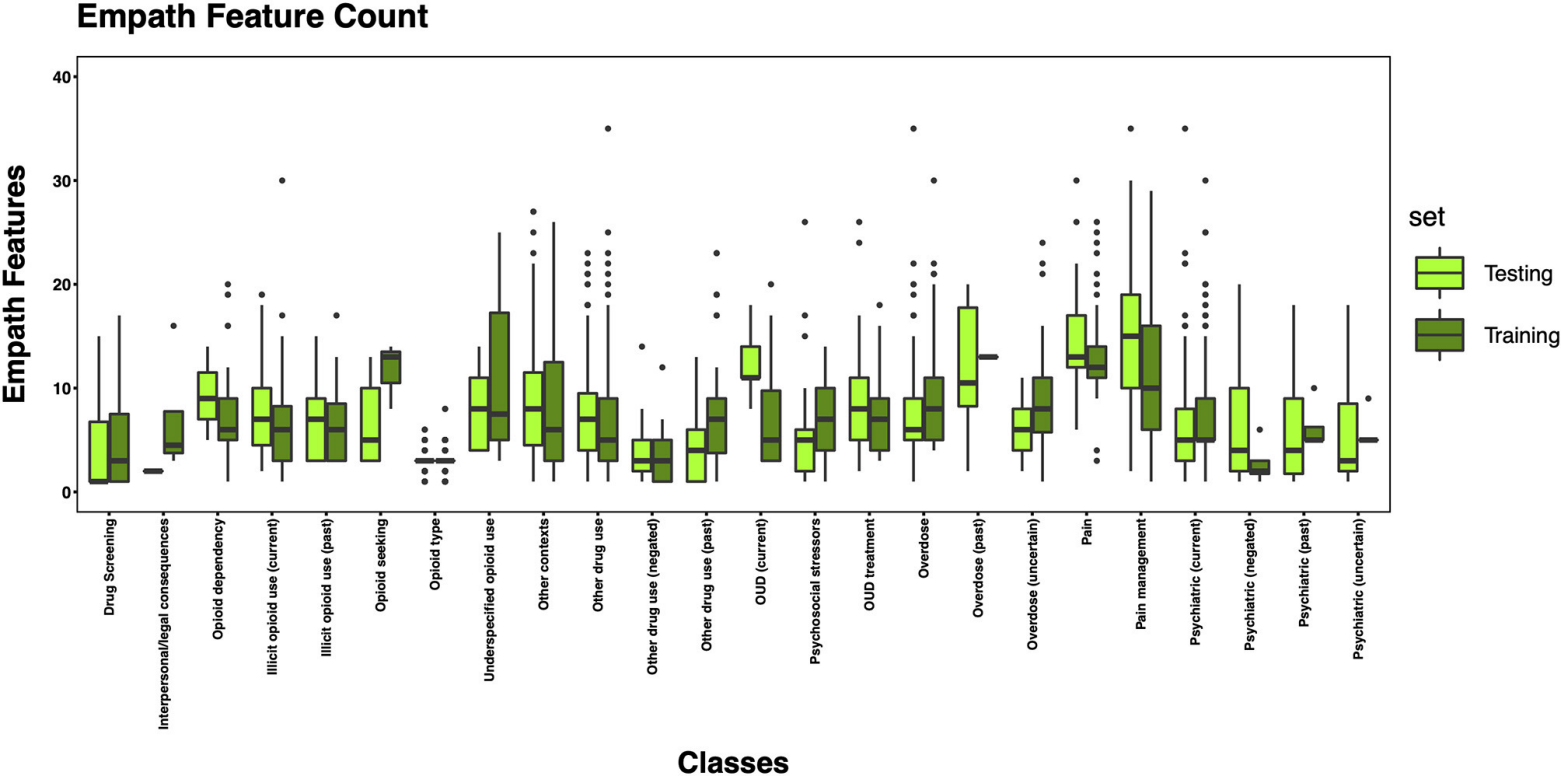
Boxplot of the mean count of Empath features per class, comparing training and testing sets.

**Figure 6:**
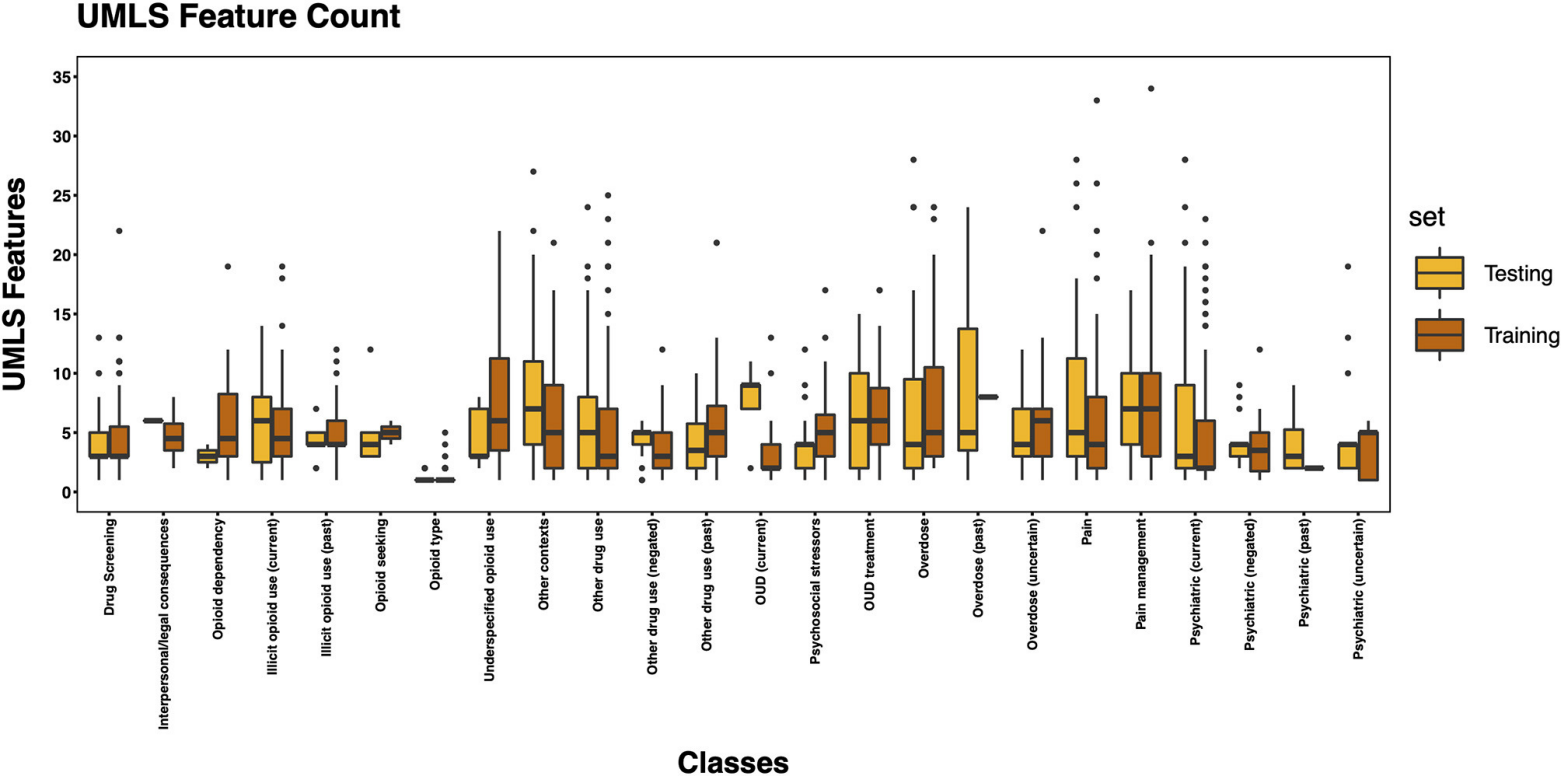
Boxplot of the mean count of UMLS features per class, comparing training and testing sets.

**Figure 7:**
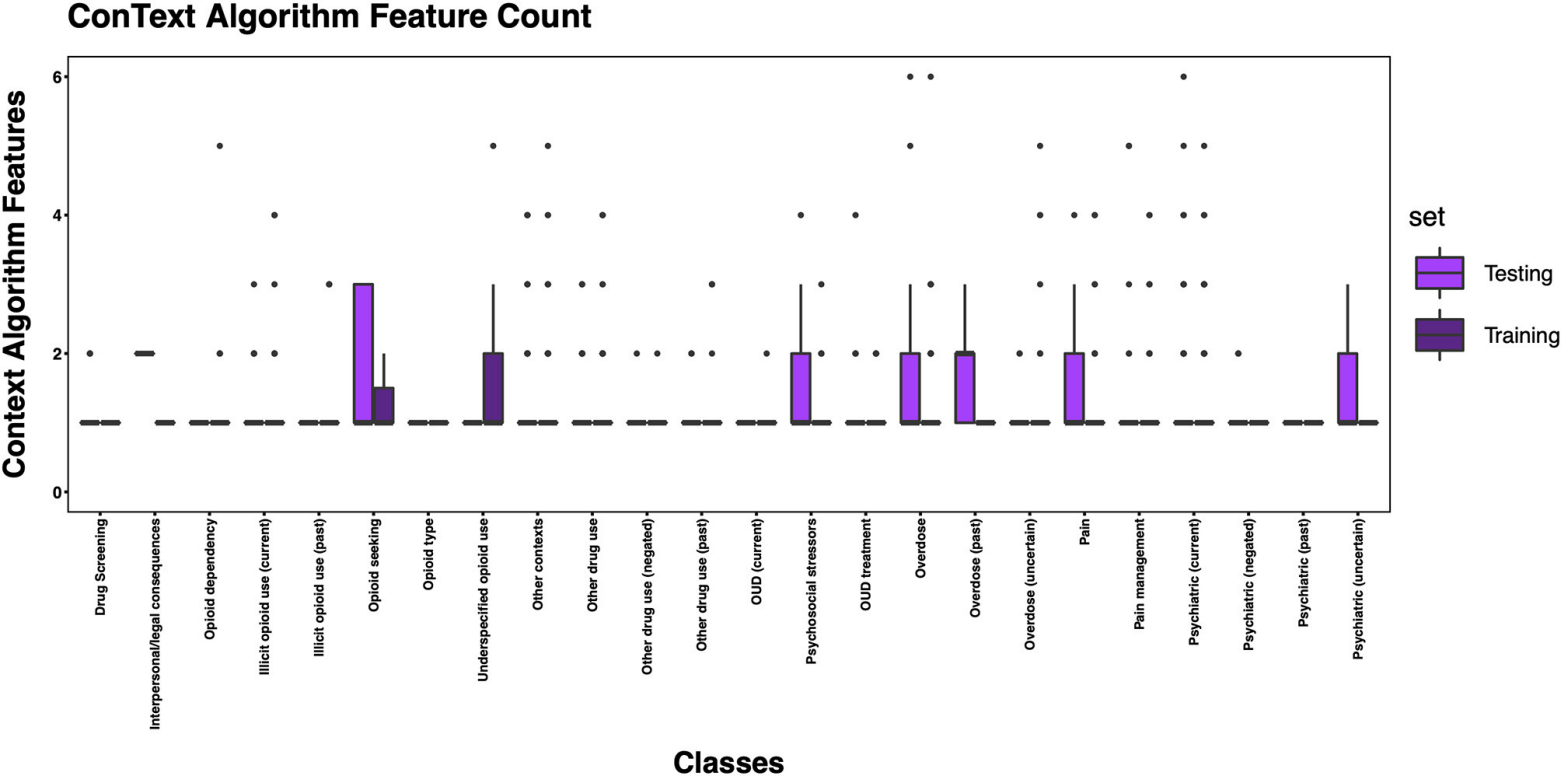
Boxplot of the mean count of contextual features per class, comparing training and testing sets.

**Figure 8:**
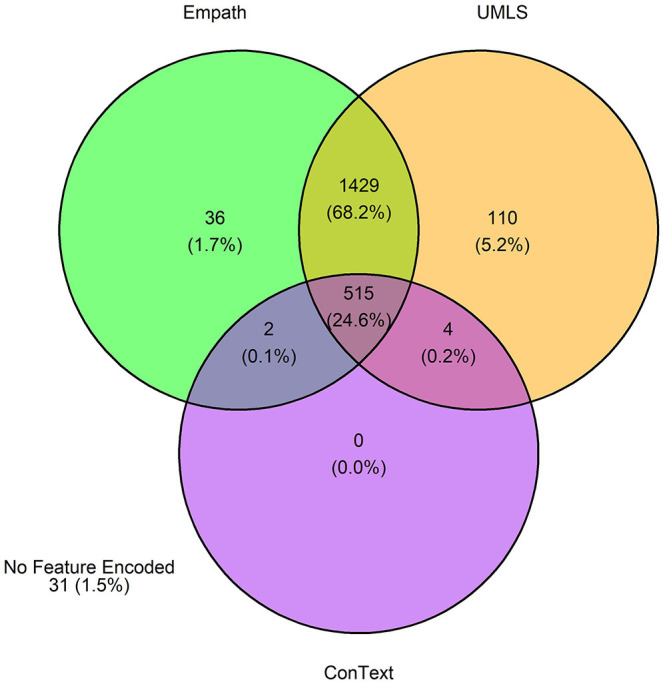
Venn diagram depicting the number of annotations in the training set encoded by each classifier.

**Figure 9:**
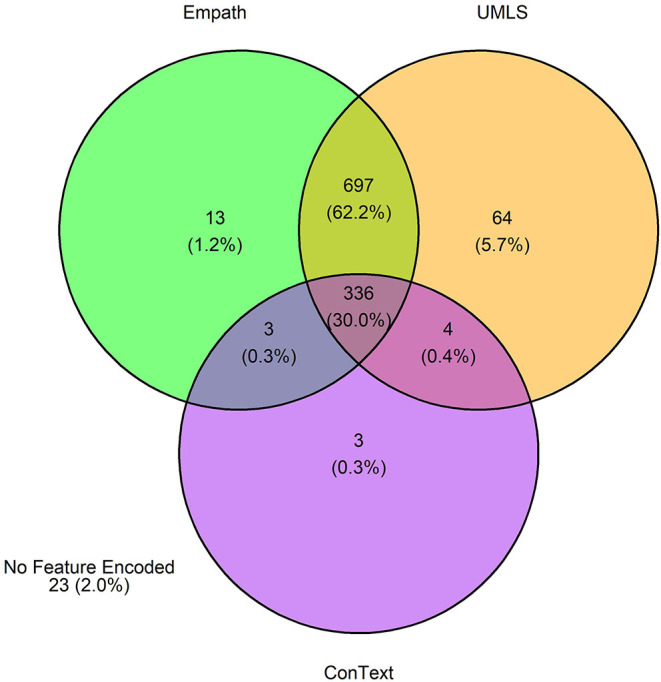
Venn diagram depicting the number of annotations in the testing set encoded by each classifier.

The 3,720 annotations were divided into training (*n* = 2,127 sentences from 66 patients) and testing (*n* = 1,143 sentences from 29 patients) datasets. Class distributions did not markedly differ between the two datasets (Figure 3). Comparing the training and testing sets pre-feature selection, the mean length of annotations in each class did not significantly differ (Figure 4), nor did the mean number of Empath (Figure 5), UMLS (Figure 6), or ConText features (Figure 7) per class. Almost all annotations (98%) were encoded by at least one feature type; 7% in both sets were encoded by just one feature type, with UMLS features being most common (Figures 8, 9).

**Figure 10:**
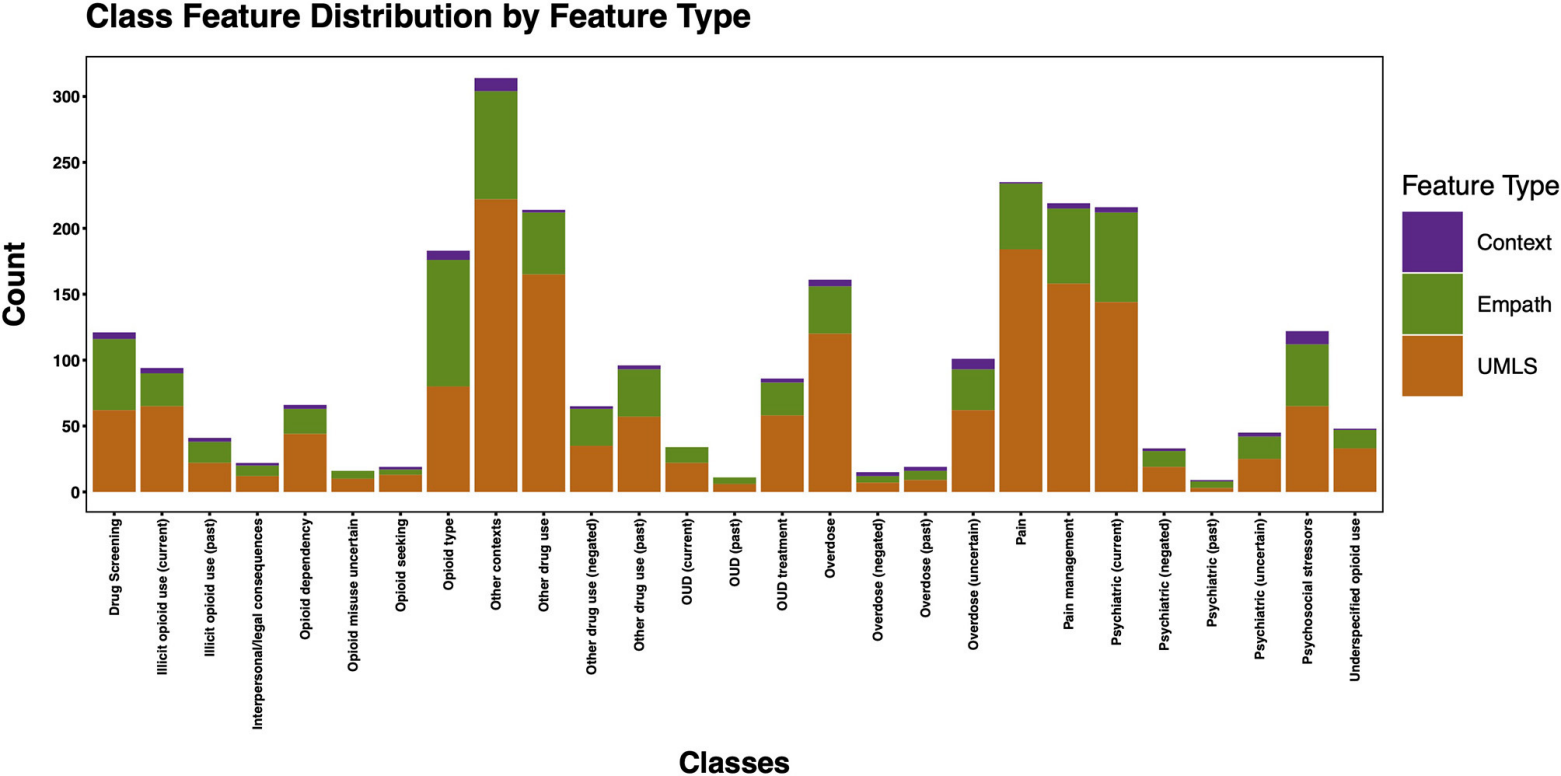
Histogram of total features per class post-feature selection by feature type.

Post-feature selection, we observed that the classes with the most features included: *other contexts, pain, pain management, psychiatric (current), other drug use*, and *opioid type* (Figure 10). The classes with the least number of features included: *psychiatric (past), OUD (past), overdose (negated), opioid misuse uncertain, opioid seeking*, and *interpersonal/legal consequences*. Across classes, the majority of features were encoded using the UMLS followed by Empath. Among the five classes with at least 100 positive annotations that were automated, we observed the following feature frequency distribution: *drug screening* (121), *opioid type* (183), *other drug use* (214), *psychiatric* (216), and *pain management* (219).

#### Sentence Classification

Among the five automated classes, we observed consistently high performance by each machine and deep learning classifier across the training and testing datasets for the following classes: *opioid type* (training F_1_: 86-96; testing F_1_: 86–99) and *drug screening* (training F_1_: 91–96; testing F_1_: 91–94) (Table 3). The highest performance was observed for Autogluon, but the two classifiers' performance was consistent for these two classes. For both classifers, we observed notable drops in F_1_ from the training and to testing set for *other drug use (current)* (−12 points for logistic regression; −17 points for Autogluon), which was driven by drops in both precision and recall. We also observed notable drops in F_1_ for *pain management* for logistic regression (−11 points), driven primarily by a drop in precision, and *psychiatric (current)* for Autogluon (−8 points), driven primarily by a drop in recall.

**Table 3:** Performance metrics (means and 95% confidence intervals) for each class with ≥100 annotation instances.

**Class**	**Training set (n** **=** **2,127;** **65% of annotated sentences)**				**Testing set (n** **=** **1,143;** **35% of annotated sentences)**			
	**Ct (%)**	**P**	**R**	**F_**1**_**	**Ct (%)**	**P**	**R**	**F_**1**_**
**Opioid type**	467 (22)				246 (22)			
Logistic regression		88 [88–88]	86 [85–87]	86 [86–87]		88 [88–88]	86 [85–87]	86 [86–87]
Autogluon		97 [97–97]	96 [96–96]	96 [96–96]		99 [99–99]	99 [99–99]	99 [99–99]
**Other drug use (current)**	295 (14)				183 (16)			
Logistic regression		66 [64–67]	44 [43–46]	52 [51–53]		51 [50–53]	36 [35–36]	40 [40–41]
Autogluon		73 [72–74]	58 [57–59]	65 [64–66]		61 [59–63]	43 [41–45]	48 [47–49]
**Pain management**	217 (10)				57 (5)			
Logistic regression		88 [88–89]	62 [61–63]	72 [71–73]		78 [76–80]	60 [58–63]	61 [60–63]
Autogluon		80 [80–80]	79 [78–80]	78 [77–79]		72 [70–74]	86 [84–88]	78 [76–80]
**Drug screening**	175 (8)				110 (10)			
Logistic regression		97 [97–97]	86 [85–87]	91 [91–91]		98 [97–98]	86 [85-87]	91 [91–91]
Autogluon		97 [97–97]	96 [96–96]	96 [96–96]		93 [92–94]	96 [95–97]	94 [94–94]
**Psychiatric (current)**	171 (8)				89 (8)			
Logistic regression		88 [86–91]	83 [81–86]	73 [69–77]		91 [89–93]	61 [60–63]	72 [70–74]
Autogluon		83 [82–84]	79 [78–80]	80 [79–81]		82 [80–84]	65 [64–66]	72 [71–73]

*Ct, count; R, recall; P, precision; F_1_, F_1_-score*.

## Discussion

In this study, we developed an annotation schema for clinical information extraction for identifying and deeply characterizing OUD based upon DSM-5 criteria for OUD, but that iteratively expanded upon these criteria to capture additional clinical, behavioral, and environmental factors related to problematic opioid use. We then automated this schema, generating and applying selected features from three NLP algorithms/knowledge sources. Using one machine learner and one deep learning approach, we trained and tested prediction models for OUD classification for the most commonly annotated classes, yielding promising results for some classes and providing direction for future study.

### Annotation Study

Using duplicate annotators for corpus 1, we achieved low overall IAA, with higher agreement for particular classes. IAA was constrained by two factors. The first factor was the infrequence of some classes (e.g., *interpersonal and legal consequences* of opioid misuse). IAA would likely increase with more instances of such signs, which may be more common in the outpatient setting as opposed to the inpatient setting captured with MIMIC III data. Thus, we believe several of these infrequent classes will remain relevant when annotating outpatient data in future work.

The second factor constraining IAA was the ambiguity of some annotation classes, which also highlights our inability to measure the validity of our annotations. This is a common problem when using pre-existing textual data, as the true intent of the writer is unknown and therefore cannot be measured against the annotation results (31). Ambiguity was most evident among classes related to potential opioid misuse. Discharge summaries were often unclear as to whether patients' use of a particular prescription opioid was legitimate (i.e., used as prescribed), misused (i.e., prescribed but not used as directed), or illicit (i.e., recreational use of non-prescribed drugs). Terms such as “narcotic” (which generally refers to opioids but can be used more broadly) added further uncertainty. Similarly, the class *other contexts* had low IAA, which is not surprising given its use as a catch-all for potentially relevant descriptions and information on contextual factors. In contrast, IAA was high for classes with low ambiguity such as *overdose, psychiatric disorder, drug screening*, and *opioid type*. The patient-level assertion for each discharge summary regarding OUD status based on the clinical writer's assertion (positive, negative, uncertain, not-specified) was not found to be useful, because virtually no discharge summaries included sufficiently definitive text that could be considered an OUD assertion. Most discharge summaries were therefore annotated as “not-specified.” Given the ambiguity of some classes in the annotation schema, we plan to make several changes to the schema for use in future work, including more nuanced classes of reported opioid use and misuse and classification of relevant concepts identified from the class *other contexts*.

### Automation Study

#### Feature Generation and Selection

We generated training and testing sets with comparable distributions by class and feature types. However, some classes contained more features post-feature selection, in part due to a limited number of annotated sentences associated with the class, but also suggesting variable semantic concepts conveyed within the annotated sentences associated with these classes. This is not surprising given that, for example, *other contexts* encoded a wide array of subtopics, (e.g., altered mental status, experiencing hallucinations, recommendations for psychiatry consultation, referral for substance use disorder treatment). Some of the classes with fewer features included greater contextualization (e.g., past, negated, current), but were not common enough to automate in this pilot study. Overall, the frequency distribution of features is correlated to the size of the vocabulary of the feature type, with a larger vocabulary resulting in a greater frequency of features. Thus, it is unsurprising that we observed more UMLS and Empath features than ConText features, as ConText captures a limited, though important, set of semantic concepts.

#### Sentence Classification

We observed sufficient annotations in the training sets to automate five classes. Among these classes, we observed consistently high performance for both logistic regression and Autogluon for two classes: *drug screening* and *opioid type*. Sentences from these classes had low lexical variability. For example, for *drug screening*, sentences often had a syntax of < drug_name>- < status>, as in “bnzodzpn-pos,” *Opioid type* was the only class not annotated at the sentence-level, instead capturing a short list of opioids such as morphine, oxycodone, and methadone, and it had the most annotations.

The class with the poorest performance was *other drug use (current)*, driven by drops in both precision and recall. In contrast with the highest performing classes, this class had much more lexical variability, ranging from short phrases such as “polysubstance abuse” to descriptive sentences such as “He formerly drank a 6-pack/day and now cut back to 3–4 beers/day,” and included a wide range of substances. Such variability may have negatively impacted precision. Acronyms and abbreviations were also problematic for this class. For example, “EtOH” and “mja,” common short terms for alcohol and marijuana, respectively, were not encoded by the UMLS dictionary, resulting in reduced recall.

In general, drops in performance for training vs. testing sets could be explained by variable statements and a small sample of training cases. By increasing the number of annotated sentences in our training set, we could improve overall performance. To improve the recall of our classifiers, we could also generate custom concept dictionaries to encode and standardize these features. For example, even with high performance for *opioid type*, we observed missing classification for some common opioids (e.g., Vicodin, dilaudid, ms contin) and misspellings (e.g., “diladudid”), indicating a need for custom dictionaries of these opioids and potential misspellings in future work. Additionally, in some cases, the knowledge bases encoded incorrect semantics. For example, Empath incorrectly interpreted “change in mental status,” identifying “change” as money, and UMLS spuriously interpreted “pt expresses the wish to stop abusing illegal substances and clean up his life,” representing “wish” as 'C1423524_NCKIPSD gene,' a NCK-interacting protein with SH3 domain. To improve the precision of our classifiers, we could develop a pre-processing step to restrict the semantic groups encoded by Empath and UMLS. The UMLS semantic network provides another opportunity to improve feature predictive power by consolidating CUIs representing topics related to substance use. For example, the CUIs “C0019187_Hepatitis, Alcoholic,” “C0728899_Intoxication,” “C0001957_Alcohol Withdrawal Delirium,” “C0085762_Alcohol abuse,” “C0001962_ethanol,” etc. could be grouped into a single alcohol feature prior to retraining and retesting the classifiers.

The traditional supervised machine learner (logistic regression) with feature engineering created a reasonable baseline for classifying sentences. However, this approach required time and effort to create informative features. In contrast, for all classes Autogluon outperformed the machine learner, demonstrating that optimal performance could be achieved with minimal up-front effort. However, this method required far greater computing resources and hyperparameter optimization may still provide more fruitful results.

#### Comparison With Prior Research

To our knowledge, this study is unique in its approach to identifying and characterizing problematic opioid use using NLP. As previously noted, few prior studies on this topic have applied machine learning or deep learning approaches, and only one study described annotation efforts that preceded the application of a machine learning approach (14). From outpatient clinical notes, Lingeman and colleages identified opioid-related aberrant patient behaviors, defined as behavior suggesting loss of control of opioid use, use of illicit substances or misuse of legal substances, and emotions or strong opinions expressed by the patient in relation to opioids (14). Their approach differed from our own, in that they used the annotated opioid-related aberrant behaviors as features in their machine learning approach, in combination with features generated from external datasets including SentiWordNet (to categorize sentiment) and word embeddings. In contrast, our annotation efforts centered on creating a corpus of data on which to test features generated by the three feature encoders (Empath, UMLS, ConText). Thus, our findings are not directly comparable. However, they found that their “hand-crafted” features had strong performance when combined with sentiment information (using SentiWordNet), suggesting the potential utility of adding sentiment features to our approach in future work.

#### Strengths and Limitations

Strengths of this study include our rigorous annotation process, the development of an annotation schema that deeply characterized OUD and related factors, inclusion of a sample population that was not limited to patients using chronic opioid therapy, the use of multiple knowledge bases to generate semantic features, and comparison of automation results from a traditional supervised machine learning system with a newer deep learning approach. This study also had limitations that should be considered when interpreting the findings. Patient data used for the annotation work was obtained solely from hospital admissions. This has implications for the representativeness of study individuals as well as for the clinical notes that we annotated. Study individuals likely had more severe health conditions and events (such as overdose) than would be expected from a general patient population. The discharge summaries that we annotated may differ qualitatively from clinical notes found in other settings. Annotation of clinical notes from other settings, such as outpatient settings, would enrich our findings. Furthermore, with patient data obtained from a US hospital, the generalizability of our findings to non-US settings is uncertain. Internationally, clinical documentation practices related to OUD may diverge, given differences across countries in opioid availability and use, opioid prescribing privileges, medication regulations, overdose management, OUD diagnostic criteria, reporting or recognition of OUD, treatment availability, stigma, and cultural factors (32, 33). Additionally, most of the study individuals had only one discharge summary and medical history was sometimes limited if a patient had no prior record within the MIMIC-III dataset. The lack of documentation of medical information over time limited our ability to identify and characterize OUD. Characterization of OUD using EHR or medical claims data is also limited by the absence of substance use severity metrics that are traditionally captured during a clinical interview to determine OUD using DSM criteria. Finally, automation was limited to five of the 32 classes due to insufficient annotations, indicating a need for a more expansive set of annotated sentences. In future work, we will expand our annotation efforts to include clinical notes from outpatient and other settings.

## Conclusions and Future Directions

In this study, we developed a rich semantic annotation schema for deeply characterizing OUD and related factors and demonstrated promising results when automating the classification. Our next steps toward development of an NLP tool to identify and characterize OUD from EHR data includes refinement of our annotation schema, as described above, and application of the schema to a larger patient sample. To overcome the limitations described, this work will utilize a patient population from an integrated health system, which will provide notes from a variety of settings (outpatient, inpatient, emergency department) across time.

## Data Availability Statement

The raw data supporting the conclusions of this article will be made available by the authors, without undue reservation.

## Author Contributions

MP contributed to data collection, analysis, and interpretation and wrote the manuscript. PF contributed to data collection, analysis, and interpretation and edited the manuscript. VT contributed to data interpretation and edited the manuscript. AD contributed to data collection and edited the manuscript. DM led the overall study, contributed to data analysis and interpretation, and wrote the manuscript. All authors contributed to the research design and approved the final manuscript.

## Funding

MP was supported by the National Institute on Drug Abuse of the National Institutes of Health under Award Number K01DA049903. PF was supported by the Ruth L. Kirschstein National Research Award (T32 HG009495). VT was supported by the National Institute on Drug Abuse of the National Institutes of Health under Award Number R01DA044015 (PI: VT) and the Pennsylvania Department of Health. DM, PF, and AD was supported by DM's start-up funding through the University of Pennsylvania.

## Author Disclaimer

The content is solely the responsibility of the authors and does not necessarily represent the official views of the National Institutes of Health.

## Conflict of Interest

The authors declare that the research was conducted in the absence of any commercial or financial relationships that could be construed as a potential conflict of interest.

## Publisher's Note

All claims expressed in this article are solely those of the authors and do not necessarily represent those of their affiliated organizations, or those of the publisher, the editors and the reviewers. Any product that may be evaluated in this article, or claim that may be made by its manufacturer, is not guaranteed or endorsed by the publisher.
