# Supplementary material for: Classifying Characteristics of Opioid Use Disorder From Hospital Discharge Summaries Using Natural Language Processing
Source: Front Public Health. 2022 May 9;10:850619. doi: 10.3389/fpubh.2022.850619 (PMC9124945; doi:10.3389/fpubh.2022.850619)
Supplement: Supplementary file 1 [file Table_1.DOCX]

Supplementary Material

**Supplemental Table 1.** Descriptions and examples of annotation schema classes

| **Class** | **Brief description/example text** |
| --- | --- |
|  |  |
| Opioid type | Any mention of an opioid (e.g., “hydromorphone,” “methadone,”) |
| Illicit opioid use (current) | Recent history of illicit opioid use (e.g., “patient admits to using heroin”) |
| Illicit opioid use (past) | Patient has a history of illicit opioid use (“history of IV heroin use”) |
| Opioid dependency | Indication of physical dependency on opioids, including signs of withdrawal, craving, or tolerance (e.g., “patient in acute opiate withdrawal”) |
| Opioid seeking | Opioid-seeking behaviors such as reporting lost opioid pills, seeking early refills, using others’ prescriptions, etc. (“reported taking husband’s methadone for headaches”) |
| Opioid misuse uncertain | Opioid misuse possible but unclear (e.g., “question of possible heroin use prior to the accident”) |
| Underspecified opioid use | No context as to whether opioid use is as prescribed or illicit (e.g., “chronic use of opiate”) |
| OUD (current) | Clinical writer asserts patient has an OUD (e.g., “heroin addiction”) |
| OUD (past) | Clinical writer asserts patient had an OUD in the past (e.g., “history of narcotic dependence”) |
| OUD (negated) | Clinical write notes patient does not have an OUD (e.g., “no history of IVDU or opiate pain med use”) |
| OUD treatment (current) | OUD treatment including medication-based treatment, counseling, detoxification, rehabilitation (e.g., “IVDU on methadone”) |
| OUD treatment (negated) | Patient not in treatment for OUD (e.g., “offered addictions counseling but refused”) |
| Opioid reduction (successful) | Efforts to cut down, control, or wean from opioid use successful (e.g., “weaned himself down to 3-4 mg PO”) |
| Opioid reduction (unsuccessful) | Efforts to cut down, control, or wean from opioid use unsuccessful (no examples identified) |
| Psychosocial stressors | Events such as trauma, homelessness, friends/family who use drugs, etc. that have potentially triggered drug misuse (e.g., “brother with IVDU”) |
| Psychiatric (current) | Acute psychiatric or mental health condition is a factor in the visit (e.g., “suicidal ideations”) |
| Psychiatric (past) | History of a psychiatric or mental health condition (e.g., “history of anxiety”) |
| Psychiatric (negated) | Lack of psychiatric or mental health condition (e.g., “denies feeling depressed”) |
| Psychiatric (uncertain) | Clinical write uncertain about whether patient has a psychiatric or mental health condition (“psychiatry questioned the patient’s diagnoses”) |
| Pain | Descriptions of pain (e.g., “chronic back pain”) |
| Pain management | Pain management efforts (“Oxycodone 5 mg Q4H as needed for breakthrough pain”) |
| Other contexts | Any other context that may be relevant to identifying OUD (highly variable) |
| Other drug use (current) | Use of non-illicit addictive substances (e.g., tobacco, alcohol) and illicit drugs other than opioids (e.g., “cocaine abuse,” “tobacco abuse”) |
| Other drug use (past) | History of use of non-illicit or illicit addictive substances (e.g., “former heavy alcohol use”) |
| Other drug use (negated) | Patient does not use a non-illicit or illicit addictive substance (e.g., “denies tobacco, EtOH, IVDU”) |
| Overdose (current) | Overdose from an opioid or other drug (e.g., “heroin and cocaine overdose”) |
| Overdose (past) | Past overdose from an opioid or other drug (e.g., “has been hospitalized for 7 medication overdoses”) |
| Overdose (negated) | Patient did not overdose (e.g., “not consistent with a narcotic overdose”) |
| Overdose (uncertain) | Uncertain whether patient had an overdose or not (“did not respond to Narcan in the ED”) |
| Drug screening | Drug screening results by type and outcome (e.g., “toxicology screen positive for benzos and opiates”) |
| Vocational interferences | Consequences of opioid use related to patient’s employment or schooling (e.g., “she was a nurse but lost her license secondary to substance abuse”) |
| Interpersonal and legal consequences | Consequences of opioid use related to interpersonal problems (relationships) or legal issues (e.g., “was arrested last Tuesday for possession of drug”) |
| Patient-level OUD assertion | Clinical writer’s assertion regarding patient’s OUD status (positive, negative, uncertain, not specified) |
